# Associations between parental autonomy support and depressive symptoms among Chinese college students: the chain-mediating effects of mindfulness and self-esteem

**DOI:** 10.3389/fpsyg.2024.1301662

**Published:** 2024-05-08

**Authors:** Ping Tan, Ruijie Wang, Tingli Long, Yaxin Wang, Chunhua Ma, Yongfeng Ma

**Affiliations:** College of Educational Science and Technology, Northwest Minzu University, Lanzhou, China

**Keywords:** parental autonomy support, depressive symptoms, mindfulness, self-esteem, college students

## Abstract

**Introduction:**

Despite extensive research on contextual factors will relieve college students’ depressive symptoms, significant gaps remain in understanding the underlying mechanisms of this relationship, particularly through individual strength factors such as mindfulness and self-esteem. Based on self-determination theory, we explore the association between parental autonomy support and depressive symptoms among Chinese college students and whether mindfulness and self-esteem serve as mediators.

**Methods:**

A total of 1,182 Chinese college students aged 16 to 27 years (*M*_age_ = 20.33, *SD* = 1.63; female = 55.7%) participated in this research. Questionnaires pertaining to parental autonomy support, mindfulness, self-esteem, and depressive symptoms were administered.

**Results:**

The results revealed that depressive symptoms were negatively correlated with both paternal and maternal autonomy support, with mindfulness and self-esteem acting as chain-mediators. Specifically, mindfulness and self-esteem were positively impacted by parental autonomy support, whereas depressive symptoms were negatively impacted by mindfulness and self-esteem. Furthermore, paternal and maternal autonomy support significantly impacted depressive symptoms via both direct and indirect pathways. Indirect effects included three paths: mediation through mindfulness, mediation through self-esteem, and mediation through the mindfulness and self-esteem chain.

**Discussion:**

The study highlights the fundamental mechanisms that account for the association between Chinese college students’ parental autonomy support and depressive symptoms, these insights can be used to prevent and manage mental health problems among Chinese college students. For example, parents’ autonomy support can directly reduce depressive symptoms, but we can also indirectly promote college students’ mental health by emphasizing the mediation role of students’ mindfulness and self-esteem.

## Introduction

In the transitional phase of higher education, college students undergo considerable psychological and social role transitions, confronting a spectrum of stressors and challenges. These factors substantively elevate their vulnerability to depressive symptoms (Arnett, 2000; Koly et al., 2021). The protracted nature of depressive symptoms can gravely impede their well-being and career success later in life (Escobar et al., 2020; Fernandes et al., 2021). Given the detrimental ramifications of depressive symptoms across various functional domains in college students, it becomes imperative to elucidate the antecedents of these symptoms. Numerous studies have demonstrated that contextual factors (such as parental autonomy support) are beneficial to reduce individual depressive symptoms (Van der Giessen et al., 2014; Johannsen et al., 2022; Wang et al., 2024). However, the scholarly knowledge with regard to the underlying mechanism of this relationship, particularly via individual strength factors, such as mindfulness and self-esteem, is relatively limited. The present study aimed to extend past scholarship by examining how those factors mediate the relationship between parental autonomy support and depressive symptoms among Chinese college students, providing insightful suggestions on intervening and preventing those symptoms.

### Parental autonomy support

Self-determination theory (Ryan and Deci, 2000) claims that autonomy is composed of individual’s initiative, ownership, and control over actions, and that it is one of three basic psychological needs (autonomy, competence, and relatedness). Parental autonomy support is interpreted as parents valuing their children’s cognitive, emotional, and cultural needs, and their ability to overcome obstacles and challenges, or encouraging them to solve problems on their own (Grolnick and Ryan, 1989; Ryan and Deci, 2020). Previous studies have shown that when parents give children more autonomy, this can promote children’s psychosocial development, including reducing the level of depressive symptoms (Muris et al., 2004; Ma et al., 2022). Autonomy is an important developmental characteristic for college students during this period (Inguglia et al., 2015). College students may face problems pertaining to learning, social interaction, and employment, for which they need to make independent decisions. Autonomy support is therefore very important for them to make choices and decisions. Although college students may live far from their parents, they need their parents’ support, especially when making important decisions (Inguglia et al., 2015; Ma et al., 2022). Therefore, we believe that autonomy support from parents will significantly affect college students’ psychological adjustment.

In addition, previous research on the function of parental autonomy support has primarily focused on the function of parents as a whole or the participation of mothers, with little discussion on fathers (Pedersen, 2017; Liu et al., 2022). Many scholars believe that the function of mothers is greater than fathers in the process of raising children (Sadeh et al., 1993; Niemiec et al., 2006). The common view is that mothers provide care and gentleness, whereas fathers represent authority and discipline. As mothers are mainly responsible for taking care of their children and will have more time to accompany them and give them emotional support, the father is responsible for financial support, and will have less contact with the children, and not be as close (Paquette, 2004; Gežová, 2015; Zajdel, 2015; Li and Lamb, 2016). The same applies to Chinese parents. In Chinese culture, men are in charge of outside affairs and women are in charge of the home, with strict fathers and loving mothers. This means that mothers are more in touch with their children at home, with a softer approach, whereas fathers are the opposite (Li and Lamb, 2013, 2016). However, as social and economic conditions have changed, the concept of the “new father” has emerged, implying that modern fathers are more committed to the process of participating in and raising children, as well as more emotionally involved (Henwood and Procter, 2003; Wall and Arnold, 2007).

To summarize, this study implies that the maternal and paternal influence on college students’ depressive symptoms and its potential mechanism should be similar. Therefore, we propose that paternal and maternal autonomy support are negatively associated with depressive symptoms.

### Mindfulness

Mindfulness refers to a state of awareness in which an individual purposefully pays attention to the present moment and does not judge the instantaneous unfolding of experience (Brown et al., 2007). Many studies have demonstrated that perceived support can improve mindfulness (Chen et al., 2020). Furthermore, mindfulness can improve both physical and mental health by reducing stress (Desrosiers et al., 2013; Shambhu et al., 2018; Yuan and Liu, 2019). Both environmental support and depressive symptoms are associated with mindfulness, which establishes the foundation for future research on the connection between parental autonomy support and depressive symptoms. Earlier studies have found that mindfulness can act as a mediator in various psychological domains. For instance, Wilson et al. (2022) discovered that mindfulness mediates the link between environmental factors (e.g., important others autonomy support) and psychological distress; specifically, the support of “important others” can promote mindfulness, while people with higher mindfulness have less psychological distress. Therefore, we proposed that mindfulness mediates parental autonomy support and depressive symptoms.

First, the growth of mindfulness will be facilitated by parental autonomy support. Research has found that parents’ acceptance and understanding during interactions with their children are beneficial to the development of children’s mindfulness (Coatsworth et al., 2010). According to other studies, parents who understand their children’s autonomy and give them more care can improve children’s mindfulness (Williams and Wahler, 2010; Geurtzen et al., 2015). However, if parents excessively control or neglect their children and fail to pay proper attention to their needs, which may cause their children to feel deeply rejected, leading to more avoidance tendencies, preventing them from focusing on the present moment and resulting in lower levels of mindfulness (Lovallo, 2012; Khaleque, 2015). As a result, we believe that parental autonomy support is beneficial for mindfulness.

Second, mindfulness can alleviate depressive symptoms. Mindfulness was initially used to treat the physical and psychological problems of patients with chronic diseases, but it has gradually been applied to regulate the emotions of ordinary people during the development process (Kabat-Zinn, 1982). Previous research has demonstrated that mindfulness can effectively reduce negative emotions like depressive symptoms (Barrett et al., 2001; Desrosiers et al., 2013; Shambhu et al., 2018). Jain et al. (2007) conducted mindfulness training among college students and found that mindfulness exercises can reduce depressive symptom levels. Further, when mindfulness increased, depressive symptom levels decreased.

### Self-esteem

Rosenberg (1965) regarded self-esteem as a self-evaluation of one’s positive or negative sense of self-worth. Self-determination theory holds that people are naturally inclined toward psychological growth and integration; if their autonomy needs are met, their self-esteem will improve. Jansen et al. (2014) and Koka (2014) found that there is a positive correlation between autonomy support from others and individual self-esteem. Nonetheless, prior studies have demonstrated that self-esteem is negatively correlated with individual depressive symptoms, meaning that people with higher degrees of self-esteem experience fewer depressive symptoms (Leary et al., 1995; Schroevers et al., 2003; Sowislo and Orth, 2012; Orth and Robins., 2013). Self-esteem is connected with parental autonomy support and depressive symptoms, which provides a basis for understanding self-esteem as a mediator. Furthermore, Ma et al. (2022) also reported that self-esteem can serve as a mediating variable between parental autonomy support and college students’ depressive symptoms. Therefore, we propose that self-esteem mediates parental autonomy support and depressive symptoms.

First, parental autonomy support may foster self-esteem. Parenting style is an important factor influencing individual’s self-evaluation; an autonomy-supporting parenting style respects one’s autonomy and allows them to form positive self-evaluations, leading to higher self-esteem (Chan and Koo, 2008; Gong and Wang, 2021). In contrast, psychological control is positively related to low self-esteem. If parents are strict and do not understand their children, low self-esteem will ensue (Milevsky et al., 2007; Bulanda and Majumdar, 2009; Gong and Wang, 2021).

Second, self-esteem can help alleviate depressive symptoms. Previous research has shown that people with high self-esteem have a more optimistic self-evaluation and look at things with a more positive eye, thereby reducing the risk of negative emotions (Sowislo and Orth, 2012; Orth et al., 2016). Cognitive susceptibility theory proposes that the way individuals pay attention to, explain, and remember negative life experiences, can increase the tendency to experience depressive symptoms (Lakdawalla et al., 2007). People with depressive symptoms frequently experience continuing negative emotions, a relatively stable negative cognitive trait known as cognitive susceptibility, which includes thoughts of failure and worthlessness, as well as dysfunctional attitudes in their self-schemas. Such maladaptive self-schemas are central to the model (Beck, 1987; Ingram, 2003). Individuals with low self-esteem adopt negative cognitive models to cope with events. When negative events occur, maladjusted self-schemas are activated that produce negative cognition, leading to the aggravation of depressive symptoms.

### Mindfulness and self-esteem

Mindfulness and self-esteem are two concepts that have received considerable attention in the field of positive psychology. Extensive research has consistently indicated that mindfulness improves individual self-esteem (Heppner and Kernis, 2007; Keng et al., 2011; Randal et al., 2015). Rasmussen and Pidgeon (2011) surveyed 205 Australian college students about their mindfulness, self-esteem, and social anxiety. The findings indicate that a significant positive association exists between mindfulness and self-esteem, and mindfulness can predict individual self-esteem. Pepping et al. (2013) discovered that when people have a high level of mindfulness, they are more likely to pay attention to their current experiences. As a result, their critical thinking and negative views decrease, and their self-esteem rises. Michalak et al. (2011) found that mindfulness includes an attitude of acceptance toward both the outside world and oneself. Individuals with higher levels of mindfulness are more likely to detect and identify self-critical thoughts as ideas rather than facts, thus preventing the emergence of low self-esteem. Therefore, this study assumes that a positive correlation exists between mindfulness and college students’ self-esteem. And we hypothesized that parental autonomy support may have an effect on depressive symptoms via a chain-mediation of mindfulness and self-esteem.

### The present study

**Figure 1:**
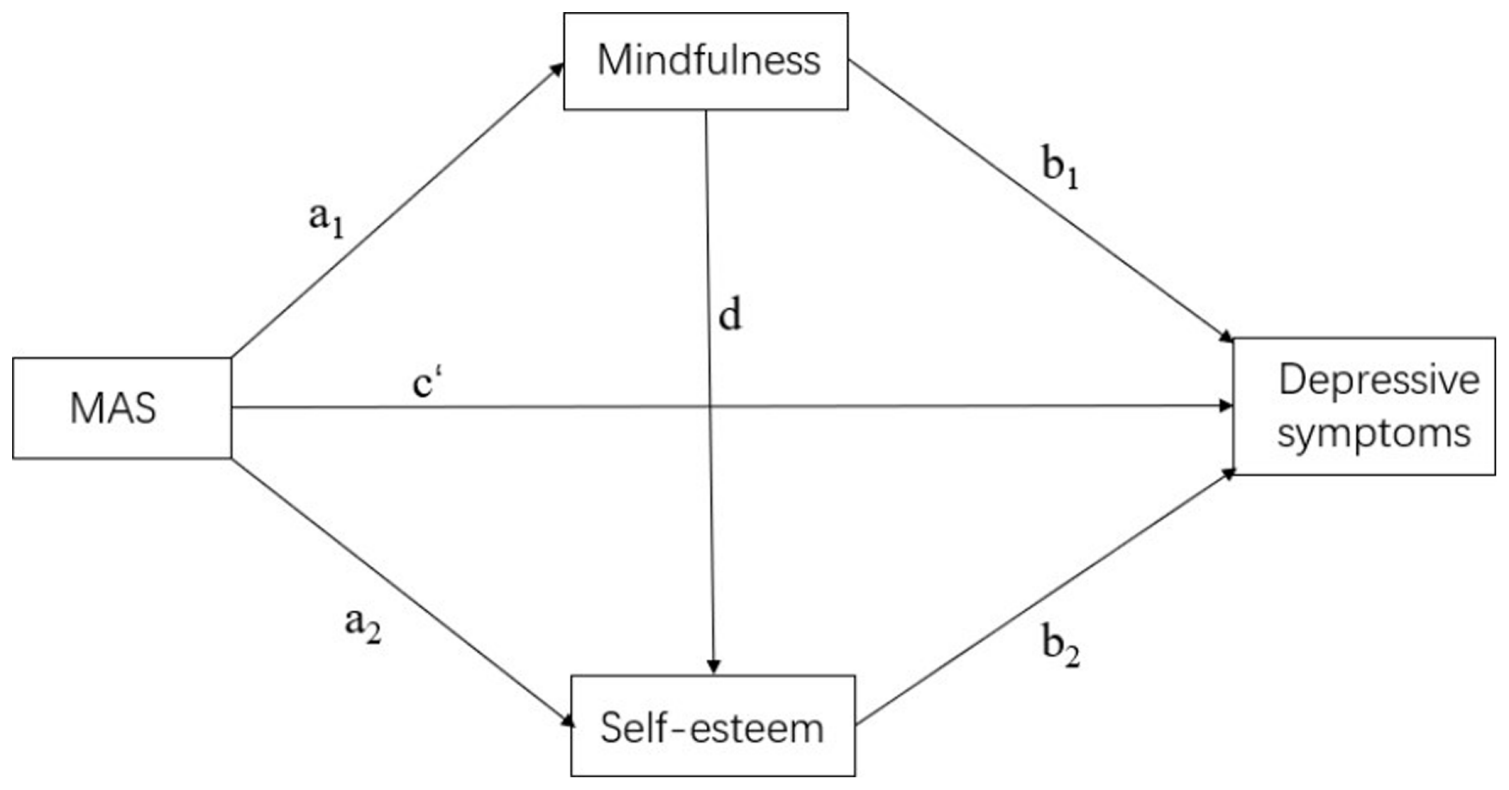
Hypothetical model. PAS, paternal autonomy support.

**Figure 2:**
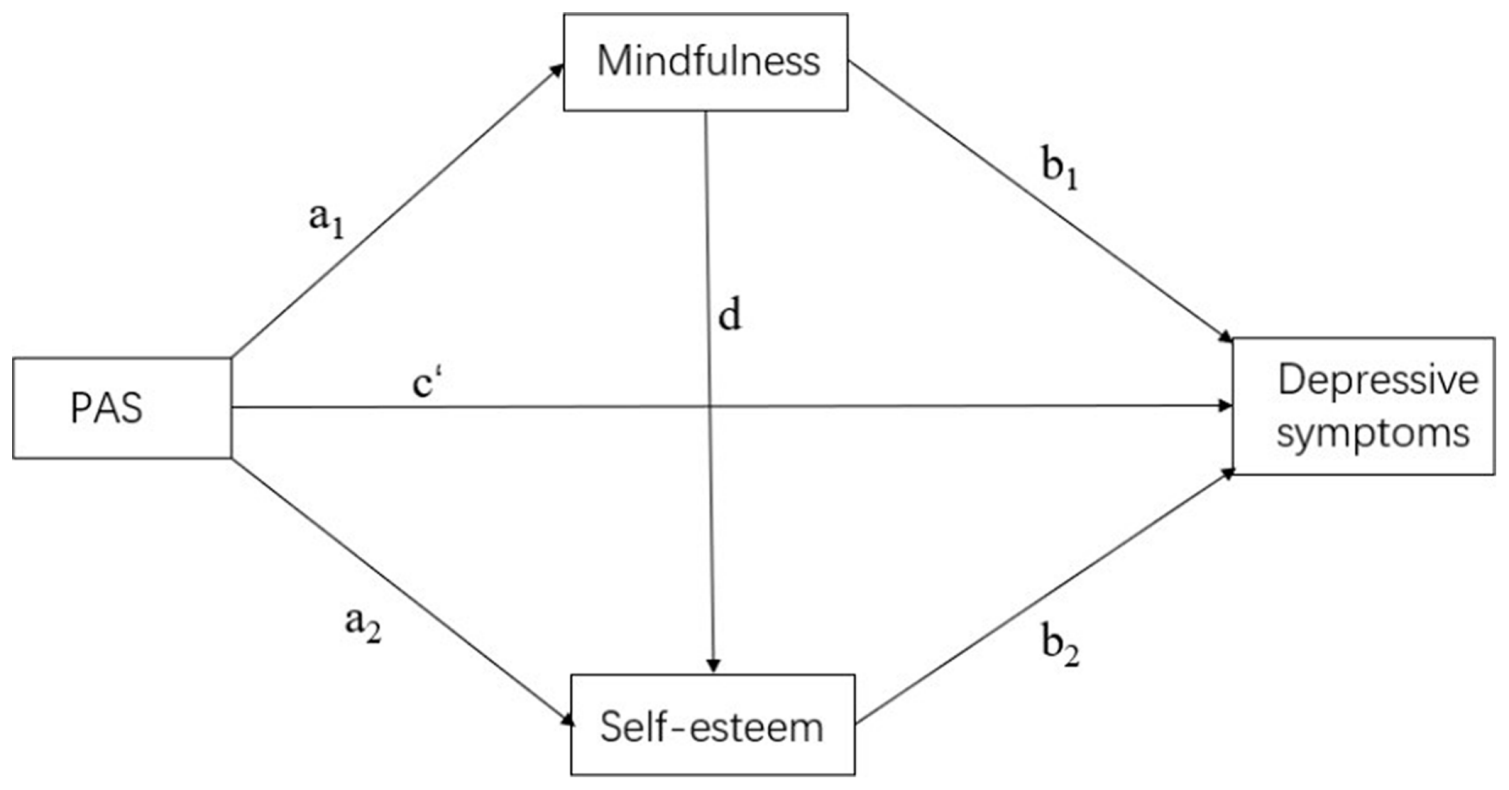
Hypothetical model. MAS, maternal support.

Based on self-determination theory, we intended to analyze the connection among paternal and maternal autonomy support and depressive symptoms, as well as explore whether mindfulness and self-esteem acted as mediating variables in this relationship (see Figures 1, 2). The following hypotheses are proposed in this study based on relevant theoretical support and previous research:

*H1*: Both paternal and maternal autonomy support are negatively related with depressive symptoms.

*H2*: Mindfulness and self-esteem mediate two relationships, including those between (a) paternal autonomy support and depressive symptoms and (b) maternal autonomy support and depressive symptoms.

*H3*: Mindfulness positively influences self-esteem, both of which act as chain-mediators between (a) paternal autonomy support and depressive symptoms and (b) maternal autonomy support and depressive symptoms.

## Methods

### Participants

The study included a valid sample of 1,182 college students. After having distributed the questionnaires online, we obtained 1,190 responses, of which eight were excluded due to repetitive answers or outliers in at least one variable (observed scores higher or lower than three standard deviations). Participants’ age ranged from 16 to 27 years (*M_age_* = 20.33, *SD* = 1.63, female = 55.7%). Based on the demographic analysis, their fathers (49.7%) and mothers (44.1%) mostly completed high school or above. Table 1 presents participants’ information in detail. Furthermore, a post-hoc power analysis was conducted. This analysis utilized an estimated effect size ranging from small to medium, in conjunction with a desired statistical power level set at 0.80 and a probability level (alpha) at 0.05. Based on these parameters, the minimum sample size required for adequate model structure analysis was determined to be 700. Our current sample size met this threshold, thereby affirming its statistical adequacy.

**Table 1:** Detailed demographic information of participants.

Variable	Classification	Frequency	Percentage
Gender	Male	524	44.30%
	Female	658	55.70%
Age	Under 18 years old	7	0.60%
	18–21 years old	902	76.30%
	22–24 years old	269	22.70%
	24 years and over	4	0.40%
Ethnological family economic income	Enjoy the local minimum living allowance	50	4.20%
	Under 3,000 RMB	153	12.90%
	3,000–5,000 RMB	344	29.10%
	5,000–8,000 RMB	149	12.60%
	8,000–12,000 RMB	199	16.80%
	12,000–20,000 RMB	174	14.70%
	More than 20,000 RMB	113	9.60%
Father’s education	Junior high school and below	595	50.30%
	Graduated from high school/technical secondary school/vocational high school/technical school.	394	33.30%
	University degree (specialized or undergraduate)	184	15.60%
	Master’s degree and above	9	0.80%
Mather’s education	Junior high school and below	661	55.90%
	Graduated from high school/technical secondary school/vocational high school/technical school.	366	31%
	University degree (specialized or undergraduate)	145	12.30%
	Master’s degree and above	10	0.80%

### Procedure

Before data collection, we obtained ethical approval from the research ethics committee of our university (the ethics permit code was XBMU 20220301). Subsequently, the author contacted two professors from two universities in Lanzhou (located in Gansu Province, Northwest China). The professors introduced the researchers and their assistants to the students and distributed QR codes among them through WeChat (a popular social networking platform in China) during class. Prior to the test, participants were informed of the study’s goals, its ethical principles, and their rights. They also provided their consent to participate in the research. All data collection and management procedures strictly followed the Declaration of Helsinki and its subsequent amendments.

### Measures

#### Parental autonomy support

The Parental Autonomy Support Scale (Wang et al., 2007) was used in this study. The questionnaire includes eight items (such as “my parents allow me to make choices whenever possible”), which were rated on a 5-point Likert scale (1 = completely disagree, 5 = completely agree), and the scale scores were averaged across all items, with lower scores indicating lower degrees of parental autonomy support. Data indicated that the scale had good internal consistency among Chinese university students (Lan and Wang, 2020). In this study, we measured the autonomy support of the mother and father separately. The Cronbach’s α coefficients of the fathers’ and mothers’ autonomy support were 0.91 and 0.92, respectively. And the CFA results of paternal autonomy support showed an acceptable model fit (χ2/df = 12.93, *p* < 0.001, CFI = 0.95, TLI = 0.94, SRMR = 0.04); the CFA results of maternal autonomy support showed an acceptable model fit (χ^2^/df = 15.7, *p* < 0.001, CFI = 0.95, TLI = 0.93, SRMR = 0.04).

#### Self-esteem

The Self-Esteem Scale developed by Rosenberg (1965) was used to measure participants’ self-esteem. The questionnaire contains 10 items (such as “I think I have many good qualities”), which were rated on a 4-point Likert scale (1 = completely disagree, 4 = completely agree). The score of the scale is the average score of all items; self-esteem levels increase with score. A prior study has demonstrated that the scale maintains adequate psychometric properties in Chinese college students (Yu et al., 2022). In the present study, the Cronbach’s α coefficient was 0.93, the results from the CFA showed an acceptable model fit (χ2/df = 26.27, *p* < 0.001, CFI = 0.89, TLI = 0.85, SRMR = 0.05).

#### Depressive symptoms

The Center for Epidemiologic Studies Depression Scale (CES-D) was created by Radloff (1977) to assess depressive symptoms among college students. The scale contains 20 items (such as “something that does not usually bother me upsets me” and “I do not want to eat; I have a bad appetite”) that are divided into four levels (not or mostly not, rarely, often, and almost always) based upon how frequently the symptoms have occurred in the past week. The sum of all question scores constitutes the scale score, with lower scores indicating fewer depressive symptoms. Findings reveal that this scale’s internal consistency among Chinese university students is acceptable (Zhang et al., 2019). Cronbach’s α coefficient was 0.84, and the results from the CFA showed an acceptable model fit (χ2/df = 5.10, *p* < 0.001, CFI = 0.95, TLI = 0.94, SRMR = 0.05).

#### Mindfulness

The Mindfulness Attentional Awareness Scale (MAAS) developed by Brown and Ryan (2003) was used in this study. The scale consists of 15 items (e.g., “I find myself not paying attention”), which were scored on a 6-point Likert scale (1 = almost always, 6 = almost never). The score of the scale is the average score of all items; a lower score indicates lower mindfulness. According to earlier research, the scale has significant reliability and validity among Chinese populations (Huang et al., 2019). In the present study, Cronbach’s α coefficient was 0.96, and the results from the CFA showed an acceptable model fit (χ2/df = 6.88, *p* < 0.001, CFI = 0.96, TLI = 0.96, RMR = 0.03).

### Data analysis

The data were analyzed using SPSS 27.0 and Amos 24.0. Firstly, we examined the descriptive information, skewness, and kurtosis of each variable. Second, we conducted Pearson’s correlation analyses and regression analysis on the variables. Third, a direct effect model about parental autonomy support and depressive symptoms was established. We measured model fit indicators such as *χ2/df*, confirmatory fit index (CFI), and normed fit index (NFI) (Hu and Bentler, 1999). If *χ2/df* is below 5 and CFI and NFI are greater than 0.9, the model has a good fit. Fourth, a sequential mediation model was established using self-esteem and mindfulness as mediating variables, and the effect values of each path were calculated using scalar and metric measures.

Finally, the bias-corrected percentile bootstrap method was applied to replicate a sample of 5,000 to estimate 95% confidence intervals (CIs) for various indirect effects. The bias-corrected percentile bootstrap method generates a new dataset by repeatedly sampling the data, and uses these data to estimate the CI of parameters. This test method corrects the estimation bias by calculating the bias of sample data, thereby estimating the CI of parameters more accurately. Additionally, it can present the CI of the mediator test under 95%, the size of the mediator effect, the significance of the direct effect and its size, and an analysis of possible missing mediators under the condition of the significant direct effect (Preacher and Hayes, 2004; Zhao et al., 2010). In this study, we used the bias-corrected percentile bootstrap method to calculate the mediation effect, and estimated the 95% CI of the mediation effect by sampling 5,000 samples. The CI did not contain 0, indicating that the effect was significant (Taylor et al., 2008).

## Results

### Descriptive statistics and correlation analysis

For the study variables, Table 2 offers descriptive statistics, including means and standard deviations, as well as bivariate correlations. The kurtosis and skewness values in the table are primarily between [−1 and 1], indicating that the study variables are normally distributed and can be further analyzed. Then a Pearson correlation analysis reveals that there were positive relationships between self-esteem, mindfulness, paternal autonomy support, and maternal autonomy support, while all of these had negative associations with depressive symptoms. In addition, except gender, other demographic variables (socioeconomic status and age) had no significant correlation with paternal autonomy support, maternal autonomy support, mindfulness, self-esteem, or depressive symptoms. Specifically, although there was no significant correlation between gender and paternal autonomy support, mindfulness, or self-esteem, maternal autonomy support was positively associated with gender, showing a stronger association in females than in males; while depressive symptoms were negatively associated with gender, showing a stronger association in males than in females.

**Table 2:** Descriptive statistics and bivariate correlations between study variables.

Variable	*M*	SD	Range	Skewness	Kurtosis	1	2	3	4	5	6	7	8
1. Depressive symptoms	38.08	10.17	1–4	0.80	0.39	–							
2. PAS	3.59	0.92	1–5	−0.86	−0.17	−0.53^***^	–						
3. MAS	3.63	0.92	1–5	−0.96	0.04	−0.55^***^	0.86^***^	–					
4. Mindfulness	4.31	1.09	1–6	−1.05	0.38	0.68^***^	0.56^***^	0.57^***^	–				
5. Self-esteem	2.90	0.65	1–4	−0.78	−0.00	−0.66^***^	0.68^***^	0.69^***^	0.70^***^	–			
6. SES	0.00	1.94	0–9	0.56	0.31	0.04	0.02	0.01	0.04	0.05	–	–	
7. Gender	–	–	1–2	–	–	−0.07^*^	0.04	0.06^*^	0.00	0.03	−0.08^**^	–	–
8. Age	20.33	1.63	16–27	–	–	0.02	0.02	0.01	0.03	0.03	−0.04	−0.03	–

*N* = 1,182. A coded as PAS, paternal autonomy support; MAS, maternal autonomy support; SES, socioeconomic status; In the gender column 1 = male, 2 = female; ^*^p < 0.05, ^**^p < 0.01, ^***^p < 0.001.

### Path analysis

Before adding the mediation variables (mindfulness and self-esteem), we analyzed the influence of paternal autonomy support and maternal autonomy support on college students’ depressive symptoms. After controlling for socioeconomic status, gender, and age, we determined that the fit index for paternal autonomy support was good: *χ2/df* = 2.16, RMSEA = 0.03, CFI = 0.98, TLI = 0.97, RMR = 0.06; the fit index for maternal autonomy support was also good: *χ2/df* = 2.63, RMSEA = 0.04, CFI = 0.98, TLI = 0.96, RMR = 0.06. Both maternal (*β* = −0.55, *p* < 0.001) and paternal autonomy support (*β* = −0.53, p < 0.001) were negatively correlated with depressive symptoms (Table 3). Thus, H1 was supported. These results indicate that paternal autonomy support and maternal autonomy support are significant predictors of depressive symptoms in college students, and maternal autonomy support has a greater effect on depressive symptoms in college students than does paternal autonomy support.

**Table 3:** Regression analysis of paternal autonomy support and mindfulness, self-esteem and depression symptoms.

Result variable	Predictive variable	*R*	*R* ^2^	*F*	*β*	*t*
Depressive symptoms	Age	0.53	0.28	115.76^***^	0.03	1.24
	Gender				−0.05	−1.91
	SES				0.05	1.91
	PAS				−0.53	−21.27^***^
Mindfulness	Age	0.56	0.31	134.63^***^	0.02	0.81
	Gender				−0.01	−0.54
	SES				0.02	0.99
	PAS				0.56	23.12^***^
Self-esteem	Age	0.78	0.61	362.97^***^	0.01	0.50
	Gender				0.01	1.66
	SES				0.02	1.30
	PAS				0.42	18.92^***^
	Mindfulness				0.46	20.94^***^
Depressive symptoms	Age	0.74	0.51	237.18^***^	0.05	2.28^*^
	Gender				−0.05	−2.60^*^
	SES				0.07	3.48^***^
	PAS				−0.07	−2.64^***^
	Mindfulness				−0.42	−15.27^***^
	Self-esteem				−0.32	−10.28^***^

PAS, paternal autonomy support; SES, socioeconomic status; ^*^p < 0.05, ^***^p < 0.001.

**Figure 3:**
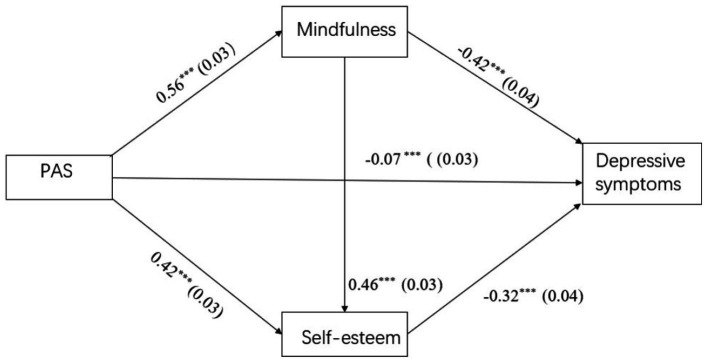
Mediation effect model. PAS, paternal autonomy support. ***p < 0.001.

**Figure 4:**
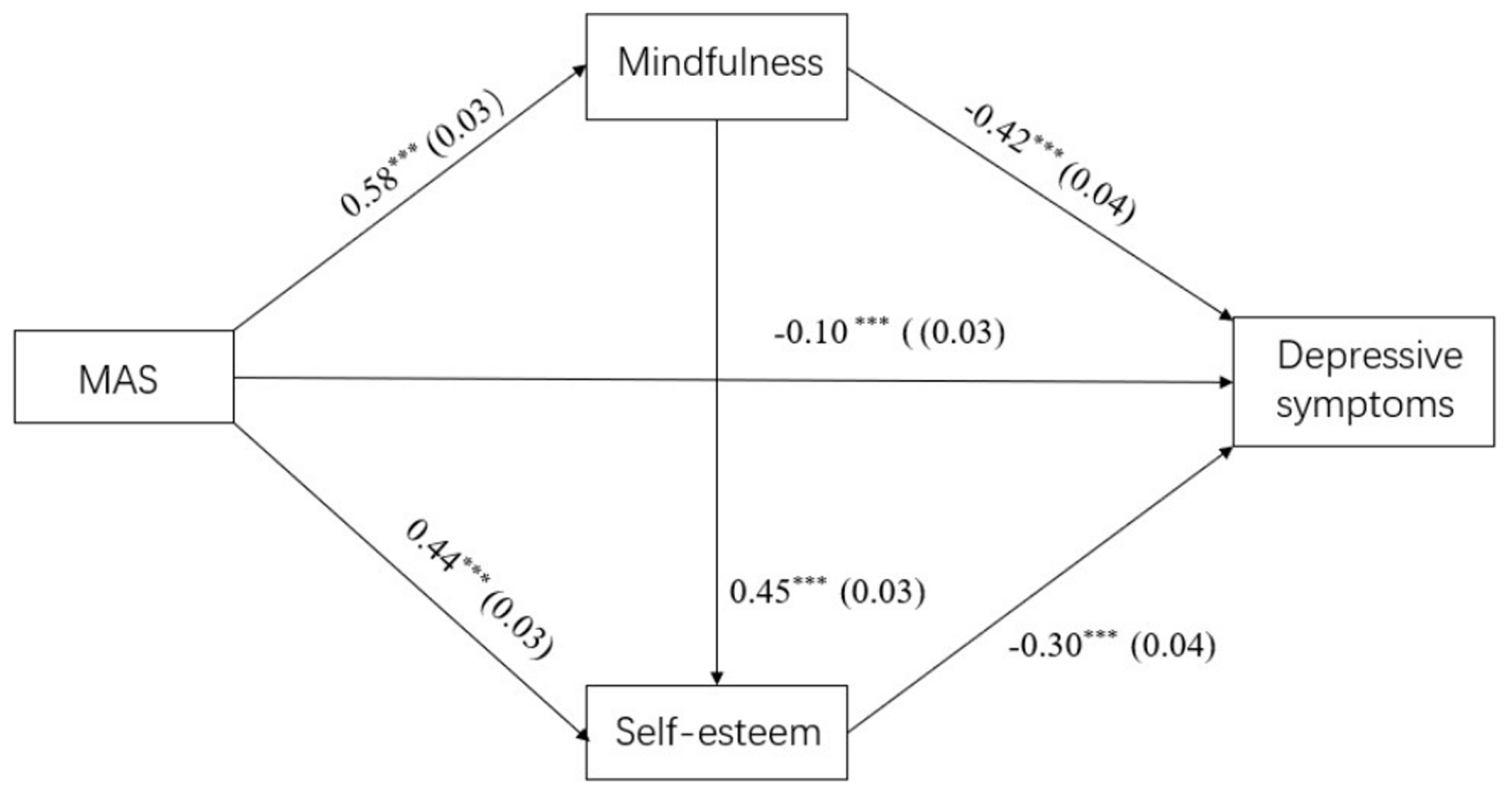
Mediation effect model. MAS, maternal support. ***p < 0.001.

With the inclusion of the mediating variables, the fit index for paternal autonomy support was: *χ2/df* = 2.16, RMSEA = 0.03, CFI = 0.997, TLI = 0.99, RMR = 0.06; the fit index for maternal autonomy support was: *χ2/df* = 2.63, RMSEA = 0.04, CFI = 0.996, TLI = 0.99, RMR = 0.06. All the load estimators were significant at the *p* < 0.01 level. Paternal (*β* = −0.07, *p* < 0.001) and maternal (*β* = −0.1, *p* < 0.001) autonomy support was positively associated with depressive symptoms; here, the direct path was significant (see Figures 3, 4 and Tables 3–5). Moreover, paternal autonomy support was positively associated with mindfulness (*β* = 0.56, *p* < 0.001) and self-esteem (*β* = 0.42, *p* < 0.001), which were negatively associated with depressive symptoms (*β* = −0.42, *p* < 0.001; *β* = −0.32, *p* < 0.001). Similarly, maternal autonomy support was positively associated with mindfulness (*β* = 0.58, *p* < 0.001) and self-esteem (*β* = 0.44, *p* < 0.001), which were negatively associated with depressive symptoms (*β* = −0.42, *p* < 0.001; *β* = −0.30, *p* < 0.001). And mindfulness (*β* = 0.46, *p* < 0.001; *β* = 0.45, *p* < 0.001) was positively associated with self-esteem (see Figures 3, 4 and Tables 3, 4).

**Table 4:** Regression analysis of maternal autonomy support and mindfulness, self-esteem and depression symptoms.

Result variable	Predictive variable	*R*	*R* ^2^	*F*	*β*	*t*
Depressive symptoms	Age	0.56	0.31	131.81^***^	0.03	1.22
	Gender				−0.03	−1.28
	SES				0.04	1.79
	MAS				−0.55	−22.72^***^
Mindfulness	Age	0.57	0.33	144.18^***^	0.02	0.88
	Gender				−0.03	−1.23
	SES				0.03	1.18
	MAS				0.58	23.93^***^
Self-esteem	Age	0.79	0.61	377.02^***^	0.01	0.59
	Gender				−0.001	−0.04
	SES				0.27	1.50
	MAS				0.44	19.85^***^
	Mindfulness				0.45	20.18^***^
Depressive symptoms	Age	0.74	0.55	239.57^***^	0.04	2.26^*^
	Gender				−0.05	−2.42^*^
	SES				0.07	3.43^***^
	MAS				−0.10	−3.68^***^
	Mindfulness				−0.42	−15.10^***^
	Self-esteem				−0.30	−9.61^***^

MAS, maternal autonomy support; SES, socioeconomic status; ^*^p < 0.05, ^***^p < 0.001.

Confidence intervals of 95% were calculated for each of the 5,000 repeated draws using the bootstrap method to measure for mediating effects. The influence of parental autonomy support on depressive symptoms was investigated through three indirect pathways (Table 5). The first indirect pathway comprised “Paternal autonomy support → Mindfulness → Depressive symptoms,” wherein the indirect effect was −0.24 and the bootstrap 95% CI = [−0.29, −0.19] excluding 0; indicating that the indirect path has a significant mediating effect. It also comprised the pathway “Maternal autonomy support → Mindfulness → Depressive symptoms,” wherein the indirect effect was −0.24 and the bootstrap 95% CI = [−0.29, −0.19] excluding 0; indicating that the indirect path has a significant mediating effect. The second indirect path comprised “Paternal autonomy support → Self-esteem → Depressive symptoms,” wherein the indirect effect was −0.13 and the bootstrap 95% CI = [−0.17, −0.10] excluding 0; indicating that the indirect path has a significant mediating effect. It also included the following pathway: “Maternal autonomy support → self-esteem → Depressive symptoms,” wherein the indirect effect was −0.13 and the bootstrap 95% CI = [−0.17, −0.10,] excluding 0; indicating that the indirect path has a significant mediating effect. The third indirect path comprised “Paternal autonomy support →Mindfulness → Self-esteem → Depressive symptoms,” wherein the effect value was −0.08 and the bootstrap 95% CI = [−0.11, −0.06] excluding 0; indicating that the indirect path has a significant mediating effect. It also included “Maternal autonomy support → Mindfulness → Self-esteem → Depressive symptoms,” wherein the effect value was −0.08 and the bootstrap 95% CI = [−0.10, −0.06] excluding 0; indicating that the indirect path has a significant mediating effect. Therefore, H2 and H3 were supported.

**Table 5:** Standardized bootstrap mediation effect test.

Variable	Route	Estimated	standard error	95%CI		*P*
				Lower	Upper	
PAS	Total effect	−0.53^***^	0.03	−0.58	−0.47	0.000
	PAS → Depressive symptoms	−0.07^***^	0.03	−0.13	−0.01	0.000
	Total indirect effect	−0.45^***^	0.03	−0.50	−0.40	0.000
	PAS → Mindfulness → Depressive symptoms	−0.24^***^	0.02	−0.29	−0.19	0.000
	PAS → Self-esteem → Depressive symptoms	−0.13^***^	0.02	−0.17	−0.10	0.000
	PAS → Mindfulness → Self-esteem → Depressive symptoms	−0.08^***^	0.01	−0.11	−0.06	0.000
MAS	Total effect	−0.55^***^	0.03	−0.60	−0.50	0.000
	MAS → Depressive symptoms	−0.10^***^	0.03	−0.16	−0.04	0.000
	Total indirect effect	−0.45^***^	0.03	−0.50	−0.40	0.000
	MAS → Mindfulness → Depressive symptoms	−0.24^***^	0.02	−0.29	−0.19	0.000
	MAS → Self-esteem → Depressive symptoms	−0.13^***^	0.02	−0.17	−0.10	0.000
	MAS → Mindfulness → Self-esteem → Depressive symptoms	−0.08^***^	0.01	−0.10	−0.06	0.000

*N* = 1,182. PAS, paternal autonomy support; MAS, maternal autonomy support; SES, Socioeconomic status, ^***^p < 0.001.

As the effects of paternal autonomy support and maternal autonomy support on depressive symptoms are considered separately, this study further conducted relative weights analysis to compare whether there are significant differences among different mediating effects (see Table 6). The comparison results of indirect effects show that there were no significant differences among the four indirect effects. Thus, the autonomy support of parents is equally important to individual’s development.

**Table 6:** Difference analysis of mediating effect.

Variable	Estimated	Standard error	95%CI		*P*
			Lower	Upper	
P_ind1_—M_ind1_	0.04	0.04	−0.03	0.11	0.271
P_ind2_—M_ind2_	0.02	0.03	−0.02	0.07	0.301
P_ind3_—M_ind3_	0.01	0.01	−0.01	0.04	0.25
P_ind4_—M_ind4_	0.08	0.05	−0.03	0.18	0.16

ind1: paternal/maternal autonomy support → mindfulness → depressive symptoms; ind2: paternal/maternal autonomy support → self-esteem → depressive symptoms; ind3: paternal/maternal autonomy support → mindfulness → self-esteem → depressive symptoms; ind4 = ind1 + ind2 + ind3.

## Discussion

Depressive symptoms are a frequently reported mood disorder among college students, which have many negative effects on their academics and future career development (Bayram and Bilgel, 2008; Pittman and Richmond, 2008). It is critical to investigate depressive symptoms among college students to assist individuals to successfully transition through this time. Under the framework of self-determination theory, this study explores the relationship between parental autonomy support and depressive symptoms of Chinese college students, as well as the role of mindfulness and self-esteem in this relationship. The findings demonstrated that paternal and maternal autonomy support were both significantly negatively related to depressive symptoms. Furthermore, mindfulness and self-esteem mediate the relationship.

The first purpose of this study was to test the relationship between parental autonomy support and depressive symptoms. The results support H1, which shows that both paternal and maternal autonomy support are negatively related with depressive symptoms. The outcome is consistent with self-determination theory as well as previous research results (Chirkov and Ryan, 2001; Wang, 2014). This indicates that both paternal and maternal autonomy support are crucial for the impact of depression symptoms in college students. The possible reason is that although traditional Chinese culture emphasizes that fathers rarely participate in raising children, with the development of the economy and the increasing proportion of women participating in social activities, the roles of men and women in the family are changing. More and more fathers are beginning to participate in parenting activities, and their role is shifting from supporting the family alone to supporting the family together (Cao and Lin, 2019; Tan, 2020). Simultaneously, Chinese fathers are increasingly recognizing their children’s autonomy, and the father-child relationship has become closer (Li, 2018). While good paternal autonomy support is a protective factor against depressive symptoms, excessive paternal intervention increases the risk of depressive symptoms among individuals (Sheeber et al., 1997; Duchesne and Ratelle, 2013). Additionally, even though college students spend more time with their peers and are far from their parents, establishing independence and autonomy is one of the most important tasks of early adulthood, and parental autonomy support is critical for passing this stage successfully (Inguglia et al., 2015).

The second purpose of this study was to explore how mindfulness and self-esteem function as mediators. Maternal and paternal autonomy support did not differ significantly; thus, we discussed them as parental autonomy support. These results suggest that the relationship between parental autonomy support and depressive symptoms is mediated by mindfulness and self-esteem. This supports H2 and confirms the results of previous research showing that parental autonomy support increases mindfulness and self-esteem, which in turn decreases depressive symptoms (Kumar et al., 2008; Du et al., 2015; Chen, 2018).

First, parental autonomy support and depressive symptoms are mediated by mindfulness. The results showed that parental autonomy support was positively associated with mindfulness. One possible reason is that parental autonomy support create a positive and supportive family environment, and individuals in autonomy-supportive environments are more likely to receive support and understanding from their parents, which helps them recognize themselves more deeply and comprehensively and cope with their own feelings with positive mental states and emotional responses (Lan and Wang, 2020; Uygun and Erus, 2024), thus making them easier to achieve and maintain a positive state of mindfulness. This is consistent with previous findings that individuals with autonomy support have more possibilities to address problems independently, are listened to and respected more often, and are more involved in planned activities or behaviors. This strong sense of engagement encourages individuals to focus on the present moment and maintain a high level of mindfulness (Coatsworth et al., 2010; Ryan and Deci, 2017). Another possible reason is that parental modeling plays an important role in cultivating mindfulness. Parents who demonstrate mindful behaviors, such as being completely attentive, actively listening, and reacting to their children without criticism, may directly impart the importance of mindfulness to their children and teach them how to engage in mindful practices. If parents can provide role models for mindfulness, their children are more likely to acquire this trait and apply it in their own lives.

Mindfulness was negatively associated with depressive symptoms. According to the basic theory of mindfulness (Brown et al., 2007), mindfulness can reduce depression by increasing an individual’s ability to maintain attention. Lu et al. (2019) found that mindfulness interventions could enhance the adaptability of Chinese youth to unfavorable environments. The fundamental reason for this is that mindfulness promotes accepting one’s inner experience and concentrating on the present moment, which allows people to become more self-aware and accepting of themselves, as well as to gain a better understanding of their own feelings, needs, and values. By accepting their own imperfections and flaws, they can easily adapt to unfavorable environments and reduce feelings of self-blame and guilt, thereby reducing depression. Mindfulness can also slow an individual’s response to stress through mechanisms that promote positive reappraisal. Higher levels of mindfulness are associated with a tendency to view problems more positively, establish positive attitudes and behaviors, and improve one’s ability to cope with setbacks and difficulties, all of which may decrease depression (Green and Kinchen, 2021; Nakamura et al., 2021). Mindfulness can reduce depressive symptoms by influencing cognitive and emotional processes. It may also decrease the risk of depressive symptoms by affecting the physiological mechanisms.

Second, self-esteem mediated the relationship between parental autonomy support and depressive symptoms. Results showed that parental autonomy support was positively associated with self-esteem. One possible reason is that self-esteem, as a form of self-evaluation, is influenced by others, especially parents (Shek et al., 2006). Parental autonomy support emphasizes the development of children’s independence, consciousness of will, and psychological freedom (Fousiani et al., 2014; Liu et al., 2023). This type of parenting can give their children full autonomy and decision-making power, and encourage them to express their thoughts and emotions. In this relationship, children are more likely to feel their parents’ inclusive and accepting attitudes, which can promote their self-affirmation, and thus achieve high self-esteem (Huang R., 2023; Huang Y., 2023).

Another possible reason is that when it comes to parenting in China, the concept of parental control may be emphasized. This is because in traditional Chinese culture, the belief of “filial piety” emphasizes the leading role of parents in the parent–child relationship, expressing the expectation that children will obey their own orders and arrangements. It is difficult for individuals receiving this kind of education to actively examine and evaluate their own abilities, and it is also difficult to establish correct and positive self-evaluation, which inevitably reduces their self-esteem level. However, with societal development and changes, the parenting style of Chinese parents has shifted from emphasizing parental control and children’s compliance to focusing on cultivating children’s independence and autonomy (Kohn, 1976; Han et al., 2021; Huang R., 2023; Huang Y., 2023). Therefore, in contemporary Chinese society, parental autonomy support also plays an important role in children’s positive psychosocial development, including the establishment of self-esteem (Ma et al., 2022).

Self-esteem was negatively associated with depressive symptoms. This indicates that self-esteem is an important internal resource that can effectively relieve the negative emotions caused by stressful events, decrease the risk of depressive symptoms, and promote mental health (Orth et al., 2012). A possible reason for this is that high self-esteem leads to a more positive and affirming attitude toward oneself, which improves one’s emotional state and decreases the risk of depressive symptoms (Wang et al., 2024). In contrast, negative self-perceptions and self-evaluations are key factors in depressive symptoms (Beck, 1987). Furthermore, individuals with high self-esteem are more likely to adopt positive and constructive coping strategies in the face of challenges (Ho et al., 2021; Hasan and Alsulami, 2024), such as seeking help and making plans, which can help them solve problems effectively and relieve depression. At the same time, they are also better at regulating their emotions and maintaining inner balance and stability; thus, parental autonomy support can also reduce depressive symptoms by increasing self-esteem.

Additionally, this study showed that mindfulness and self-esteem had a chain-mediating effect on the relationship. This finding supports H3 that parental autonomy support can indirectly affect self-esteem by promoting mindfulness and can ultimately reduce depressive symptoms among college students. One possible reason is that individuals’ own high levels of mindfulness make them pay more attention to their current experiences and adopt a non-judgmental and non-reactive attitude toward their various feelings. This allows them to observe and understand their emotions, thoughts and behaviors more deeply, leading to a fuller understanding of themselves (Michalak et al., 2011; Pepping et al., 2013). Self-awareness and acceptance are processes that help individuals establish a more positive self-evaluation, eliminate self-doubt and negative self-assessment, and ultimately increase their self-esteem (Stenhaug and Solem, 2023; Wang and Chen, 2023). Mindfulness emphasizes the cultivation of self-compassion (understanding and accepting one’s emotions and needs) and reduces self-criticism. By cultivating self-sympathy, individuals can better care for themselves, enhance their sense of self-worth, and further enhance their self-esteem (Morley and Fulton, 2019; Tran et al., 2022). Therefore, mindfulness and self-esteem play chain-mediating roles between parental autonomy support and depressive symptoms among college students.

In summary, parental autonomy support can directly reduce depressive symptoms, but it can also indirectly promote college students’ mental health by emphasizing the mediating role of students’ mindfulness and self-esteem. According to self-determination theory (Ryan and Deci, 2000), when individual needs for autonomy are met, they can act according to their wants and choices rather than being restricted by other’s expectations or external pressure. This kind of autonomous experience helps enhance an individual’s intrinsic motivation and self-efficacy (Jehanghir et al., 2023), allowing them to generate motivation to pursue their own goals. This intrinsic motivation can improve an individual’s self-awareness and attention, make the individual pay more attention to the present moment, and participate in mindfulness exercises, thereby improving mindfulness (Pan et al., 2024). When people achieve their goals, they feel a sense of achievement that enhances their self-esteem.

Additionally, in the college context, the need for competence and relatedness related to the need for autonomy (the three basic human needs) may also play a positive role in the process of mindfulness promoting self-esteem. Research has demonstrated that parental autonomy support promotes competence and relatedness (Abidin et al., 2022; Barberis et al., 2022). Competence refers to the belief that one can successfully do something. When individuals feel competent, they are more focused on the task and experience at hand rather than plagued by worry, insecurity, or self-doubt. This state of concentration allows individuals to perform better and feel a sense of accomplishment from what they are doing, resulting in a greater sense of certainty about their abilities and worth (Chen, 2024). Relatedness refers to an individual’s perception of good relationships and support from others. Parental autonomy support not only cultivates the need for autonomy but also cultivates a sense of relatedness. When individuals have a strong sense of relatedness, they are more likely to obtain support and assistance from the social context, employ good coping strategies to deal with difficulties, look at them objectively, and not deliberately avoid or magnify them (Rashid et al., 2020). In this context, individuals feel safe and can explore and accept their thoughts and feelings without judgment, which helps enhance the impact of mindfulness on self-esteem.

### Implications

Theoretically, this study discusses the protective factors that affect the depressive symptoms among college students. This enabled us to have a comprehensive and systematic understanding of the potential mechanism of parental autonomy support affecting depressive symptoms. Secondly, paternal and maternal autonomy support are discussed as independent variables, which further enriches the relevant research.

The practical implications of this research necessitate a collaborative approach between families and academic institutions in mitigating and addressing depressive symptoms in college students, with a particular emphasis on fostering mental health development. Initially, acknowledging the significance of parental autonomy support, higher education institutions should endeavor to inform parents about the criticality of this support. Special emphasis should be placed on the pivotal role of paternal involvement. This can be achieved through various platforms such as online science popularization initiatives, educational lectures, and interactive forums for discourse and exchange. Such measures aim to empower parents in endorsing their children’s autonomy, valuing their perspectives, and moderating control. Second, given the mediating role of mindfulness and self-esteem between parental autonomy support and depressive symptoms, academic institutions can proactively enhance these attributes in students. This could be facilitated through structured group counseling sessions and comprehensive mental health curricula. For instance, mindfulness training programs could be designed to assist students in acknowledging and appreciating their immediate environments. Concurrently, activities geared toward self-esteem enhancement, such as exercises encouraging students to identify and reflect on their strengths, comprehend their intrinsic value and potential, and foster accurate self-appraisal, could significantly bolster their self-esteem. These interventions collectively aim at reducing the prevalence and impact of depressive symptoms among college students.

### Limitations and future prospects

Although the present study explored the value of parents separately on their children’s mental health, it has some drawbacks. First, the research design was cross-sectional, which made it challenging to establish a strictly meaningful causal relationship. Second, the research variables were self-reported by college students, which could have caused measurement deviation. Third, although the sample size was adequate and reliability was high, this study primarily focused on one region. Thus, future research should track and measure depressive symptoms continuously to clarify the dynamic situation of individual depressive symptoms and reveal more factors affecting depressive symptoms. Further, future studies should use the multi-agent measurement method to reduce the measurement error and qualitative interview to examine the subject from another angle. Subsequent research should expand the scope and generalizability of the study by including more subjects, such as age and region. Lastly, in the future, scholars should examine parents with high education level to verify whether the difference in the level of education will affect the mediating effect of mindfulness and self-esteem.

## Conclusion

This study found that both paternal and maternal autonomy support have a significantly negative effect on depressive symptoms among college students. Further, mindfulness and self-esteem play a mediating role in this relationship. This study revealed the potential mechanism of parental autonomy support in the context of depressive symptoms and provided theoretical support for promoting mental health of college students.

## Data availability statement

The raw data supporting the conclusions of this article will be made available by the authors, without undue reservation.

## Ethics statement

The study involving humans was approved by the Research Ethics Committee of Northwest Minzu University. The study was conducted in accordance with the local legislation and institutional requirements. Since the participants were college students aged above 18, we informed the participants before the investigation that the study was anonymous, participation was completely voluntary, and they had the freedom to withdraw at any time. The investigation was performed with the consent of the participants. Written informed consent for participation in this study was provided by the participants’ legal guardians/next of kin.

## Author contributions

PT: Conceptualization, Formal analysis, Investigation, Methodology, Software, Writing – original draft. RW: Conceptualization, Investigation, Writing – review & editing. TL: Conceptualization, Investigation, Validation, Writing – review & editing. YW: Investigation, Supervision, Writing – review & editing. CM: Data curation, Funding acquisition, Resources, Supervision, Writing – review & editing. YM: Project administration, Resources, Writing – review & editing.
